# Bifunctional polymer assisted growth of crack-free thick perovskite films for flexible X-ray detection

**DOI:** 10.1039/d6tc00075d

**Published:** 2026-03-20

**Authors:** Qianrui Li, Donato Valli, Roel Vanden Brande, Giorgia Rizzi, Johan Hofkens, Wei Qu, Elke Debroye

**Affiliations:** a Department of Chemistry, Molecular Imaging and Photonics, KU Leuven Celestijnenlaan 200F 3001 Heverlee Belgium; b State Key Laboratory of Advanced Fiber Materials, Donghua University Shanghai 201620 China; c Institute of Supramolecular Science and Engineering (ISIS) Strasbourg Grand Est France; d Max Planck Institute for Polymer Research 55128 Mainz Germany; e School of Optical and Electronic Information, Suzhou City University & Jiangsu/Suzhou Key Laboratory of Biophotonics Suzhou 215104 China

## Abstract

The expanding use of perovskite materials in flexible optoelectronics has sparked growing interest in their application for flexible X-ray detectors. Achieving thick perovskite films is crucial for effective X-ray absorption; however, such films typically exhibit brittleness and crack formation. In this work, we present a bifunctional polymer-assisted approach employing the triblock copolymer Pluronic P123, composed of poly(ethylene glycol) (PEG) and poly(propylene glycol) (PPG), to simultaneously control crystallization and enhance the mechanical integrity of lead-free Cs_2_AgBiBr_6_ (CABB) thick films. The PEG segments coordinate selectively with Ag^+^ ions, guiding evolution to the stable 3D phase, while the PPG segments introduce steric effects that mitigate excessive Ag^+^ coordination and favor uniform crystal growth. This cooperative mechanism yields crack-free, highly crystalline, and mechanically flexible perovskite films. The resulting P123-modified CABB detectors demonstrate a remarkable X-ray sensitivity of 244.71 µC Gy^−1^ cm^−2^ and low detection limit of 121 nGy s^−1^ under a low bias of 50 V mm^−1^, over twice that of unmodified devices. Moreover, the modified detectors maintained over 70% of their initial sensitivity under small bending radii and over 80% after 500 bending cycles, exhibiting outstanding fatigue endurance and long-term stability over a 60-day period, in contrast to the pronounced degradation seen in pristine CABB devices. This study establishes a polymer-guided design paradigm for fabricating lead-free, flexible, and scalable perovskite-based radiation detectors.

## Abstract 2

Pluronic P123 regulates crystallization of lead-free Cs_2_AgBiBr_6_, yielding crack-free, flexible thick films. PEG–Ag^+^ coordination and PPG steric effects enable uniform growth. Devices show high sensitivity, low detection limit, strong bending durability, and long-term stability.

## Graphical abstract

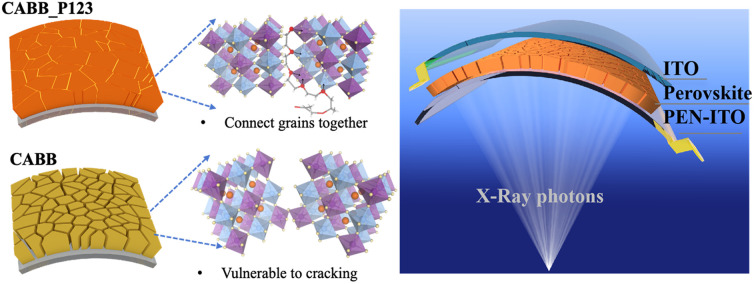

## Introduction

Flexible X-ray detectors are attracting growing interest for wearable health diagnostics, portable medical imaging, and compact security screening, where flexibility and conformability to curved surfaces are essential. Compared to rigid detectors, flexible detectors enable direct integration onto skin, clothing, or non-planar equipment surfaces, thereby enabling real-time, continuous, and spatially resolved monitoring.^1–4^ These factors impose stricter requirements on the photosensitive layer, such as mechanical durability and environmental stability. Among various candidates, lead-free halide perovskites have emerged as a promising class for X-ray detection due to their low toxicity, high X-ray absorption coefficients enabled by heavy elements, long carrier diffusion lengths, and low-temperature solution processability suitable for large-area, flexible, and cost-effective fabrication.^2,5–11^ However, efficient X-ray attenuation typically relies on thick perovskite layers ranging from tens to hundreds of micrometers, to ensure sufficient absorption of high-energy photons. Meanwhile, sub-micrometer perovskite films have been demonstrated to deliver high X-ray sensitivity and flexibility in self-driven detection architectures.^12,13^ This fundamental trade-off between absorption efficiency and mechanical compliance poses a key challenge for flexible X-ray detectors, as increasing thickness inevitably exacerbates stress accumulation and grain boundary weakness, leading to cracking and delamination under mechanical deformation.^4,10,11,14,15^

Numerous studies have explored a wide range of additive and encapsulation strategies to address the mechanical or thickness issues of perovskite film. For example, perovskite-filled membranes fabricated through repeated vacuum infiltration enable flexible, large-area X-ray detectors with excellent bendability; however, such performance relies on technically demanding, multi-step processing conditions that are challenging to scale.^16^ The use of polymer additives has also been widely investigated to improve the microstructure and stability of halide perovskite films. The long-chain poly(ethylene glycol) (PEG) increases solution viscosity and supplies additional nucleation sites, enabling thicker films with improved coverage.^17,18^ Polyurethane^19^ and polyvinylpyrrolidone (PVP)^20^ have been incorporated to enhance grain size, moisture tolerance, and defect passivation of thin (<1–2 µm) perovskite layers. Recently, polyimide (PI)-induced layered polymer–perovskite architecture can impart brittle perovskites with plastic-like flexibility and fatigue tolerance while retaining electronic transport.^21^ In addition, small-molecule crosslinkers have been shown to improve the flexibility of perovskite films by passivating surface and interfacial defects.^22–24^ These strategies have revealed key principles of nucleation control, organic–inorganic interactions and mechanical compliance enhancement that provide a valuable foundation for extending additive engineering toward thicker, flexible perovskite architectures.

Herein, we propose a dual-functional additive strategy based on Pluronic P123, a triblock copolymer composed of poly(ethylene glycol) (PEG) and poly(propylene glycol) (PPG), to simultaneously regulate the crystallization kinetics and improve the mechanical flexibility of lead-free Cs_2_AgBiBr_6_ (CABB) films. The PEG segments in P123 selectively coordinate with Ag^+^ ions, effectively regulating the phase transition from layered intermediates to the stable CABB structure, while the hydrophobic PPG segments suppress excessive Ag^+^ coordination, thereby enabling more controlled and uniform crystallization. Meanwhile, the P123 chains serve as polymeric binders, enhancing crystallite connectivity and film flexibility. The integrated polymeric architecture enables a synergistic modulation of nucleation dynamics, film morphology, and mechanical robustness, which not only facilitates the fabrication of flexible, uniform, and defect-suppressed thick CABB films, but also effectively suppresses crack formation during ambient-condition fabrication without requiring strict thermal annealing. Moreover, devices based on P123-modified CABB films exhibit an even improved X-ray sensitivity of 244.71 µC Gy^−1^ cm^−2^ compared to the pristine microcrystalline material, low detection limit of 121 nGy s^−1^, high mechanical resilience under repeated bending, and excellent long-term operational stability. These results highlight the potential of scalable copolymer-assisted processing to address the dual requirements of X-ray absorption and mechanical flexibility in eco-friendly, lead-free perovskite systems.

## Results and discussion

### Design strategy and synthesis

All-inorganic halide perovskites such as CABB offer high chemical stability and intrinsic environmental robustness.^25^ However, their brittle nature poses significant challenges for applications requiring mechanical compliance, such as flexible or large-area X-ray detectors.^20,21^ To address this, we adopted a strategy of polymer-assisted crystallization and embedding, aiming to improve both the microstructural integrity and mechanical adaptability of thick perovskite films.

**Fig. 1:**
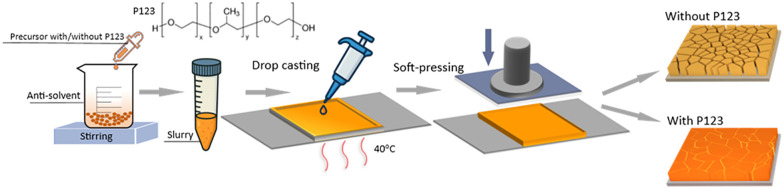
Schematic diagram of the preparation of CABB films with/without P123, insets show the P123 structure.

Amphiphilic block copolymers combine distinct chemical segments capable of interacting with both inorganic precursors and the evolving structure.^26^ We focused on Pluronic triblock copolymers (PEG–PPG–PEG) with varied PEG/PPG ratios and molecular weights. PEG alone enhances flexibility and defect passivation through strong coordination with metal ions, whereas PPG promotes crystallization owing to its hydrophobic backbone and weaker coordination ability. Among them, Pluronic P123 was selected for its moderate molecular weight (*M*_n_ ≈ 5800 g mol^−1^), balanced hydrophilic–hydrophobic character (PEG : PPG = 3 : 7 by weight), and excellent solubility in polar solvents and high solubility in polar solvents.^22^ Unlike tailor-made polymer systems that often require complex synthesis and purification, P123 is commercially available, inexpensive, and compatible with ambient, low-temperature fabrication. As shown in Fig. 1, we synthesized CABB microcrystals by applying a previously reported anti-solvent method.^23^ Pluronic P123 (10 wt%) was added to the precursor containing BiBr_3_, CsBr, and AgBr in DMSO and stirred for 2 h to form a homogeneous solution. The resulting precursor (with or without P123) was then dropwise added into isopropanol at 60 °C for 2 h, yielding CABB and CABB_P123 slurry precipitates. The films were fabricated by drop-casting the centrifuged the perovskite slurry into a square template (thickness 0.5 mm) on pre-cleaned ITO–PEN substrates maintained at 40 °C, followed by soft pressing to ensure film uniformity and adhesion.^27^

### Crystallization and morphological evolution induced by P123

**Fig. 2:**
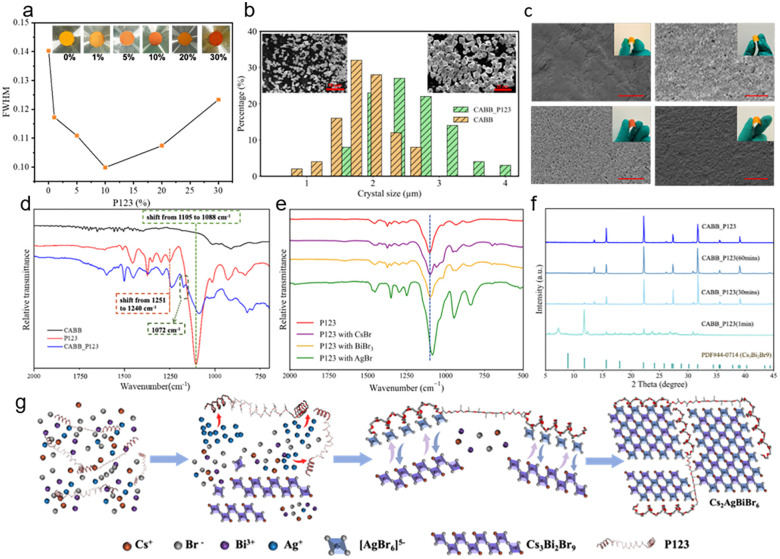
(a) FWHM of the [220] diffraction peak. (b) Crystal size distribution of pristine CABB and CABB_P123 powders. Insets show the corresponding SEM images. Scale bar = 10 µm. (c) SEM images of film surfaces after mechanical bending for CABB, CABB_PEG, CABB_PPG, and CABB_P123 samples. Scale bar = 50 µm. Insets display optical images during bending tests. (d) FT-IR spectra of pure P123, CABB, and CABB_P123. (e) FT-IR spectra of P123 reacted with CsBr, BiBr_3_, AgBr. (f) XRD patterns of CABB_P123 films at various intermediate stages during film formation. (g) Schematic illustration of the crystallization.

The incorporation of Pluronic P123 significantly enhances the crystallinity and thickness of CABB layers as confirmed by X-ray diffraction (XRD) and scanning electron microscopy (SEM). As shown in Fig. S1, all samples maintain the pure CABB phase,^24^ indicating that P123 does not change the crystal structure. With increasing P123 concentration, the diffraction peaks of perovskite CABB become sharper, the full width at half maximum (FWHM) of XRD peaks especially at 22.3° and 31.7° narrows, reaching a minimum at 10 wt%, suggesting higher crystallinity (Fig. 2a and Fig. S1b, c, and d). In addition, the color of CABB powders gradually shifted from yellow to orange suggesting enhanced crystallite size and improved crystallinity, which can be attributed to the transformation of incomplete crystal growth towards more uniform toward larger, well-defined grains. Fig. 2b shows the SEM images and corresponding crystal size distribution of CABB and CABB_P123. Pristine CABB crystals are predominantly distributed within the 1.0–2.2 µm range. Upon modification with P123, the perovskite crystals exhibit significantly larger sizes range, increasing to 2.0–3.0 µm, together with well-defined faceted shapes and improved uniformity. However, excessive P123 loading (≥20 wt%) leads to morphological irregularities and secondary phases (Fig. S1a and S2), in agreement with the broadened XRD reflections and increased FWHM observed in Fig. 2a, highlighting 10 wt% as the optimal concentration for achieving beneficial structural improvements. This optimized sample is referred to as CABB_P123.

Consistent with these structural improvements, UV–vis absorption spectra in Fig. S3 exhibited an obvious redshift in the absorption edge, while Tauc plot analysis confirmed a corresponding bandgap narrowing from 2.24 eV (pristine) to 2.09 eV upon adding 10 wt% P123. This bandgap reduction is attributed to suppressed quantum confinement effects due to crystallite growth.^28,29^ The optical and morphological changes show that P123 promotes the growth of larger, well-ordered perovskite crystals, with 10 wt% P123 yielding the optimal balance of size, uniformity, crystallinity, and phase purity for high-quality CABB films.

To better understand the underlying mechanism responsible for these structural improvements, both pure PEG and pure PPG were introduced into the CABB precursor at the same concentration (10 wt%) and under identical processing conditions as P123. Thick films of CABB, CABB_P123, CABB_PEG, and CABB_PPG were made *via* drop casting, followed by mild annealing and soft pressing. SEM images in Fig. 2c reveal that P123-assisted films exhibit a dense and uniform morphology, in contrast to the rough and pinhole-rich surface observed in control samples. Morphology analysis by SEM (Fig. S4) reveals that solely PEG incorporation results in overgrown, irregular crystals, and the corresponding CABB_PEG layers show a secondary Cs_3_Bi_2_Br_9_ phase (Fig. S5) and poor film quality (Fig. 2c). In contrast, CABB_PPG layers exhibit sharp and well-defined XRD peaks without impurities (Fig. S5) and SEM reveals uniform microcrystalline films with enlarged crystallites (Fig. 2c). These features indicate that the hydrophobic PPG acts primarily through steric hindrance, reducing the number of nucleation sites and favoring the growth of fewer but larger crystals. Such a mechanism is in line with previous findings by Huang *et al.*, who reported that moderate surface hydrophobicity can promote slow, homogeneous nucleation in perovskite systems.^30^ Nevertheless, despite the improved crystallinity, CABB_PPG films are brittle and prone to cracking especially after bending (Fig. S6), highlighting that steric control alone fails to provide mechanical compliance, whereas the CABB_P123 films maintain structural integrity under deformation. These control experiments emphasize that the synergy of PEG and PPG within P123 is required to simultaneously achieve high crystallinity and flexibility.

FT-IR spectroscopy provides additional insights into the interactions between the polymer and the perovskite lattice. As shown in Fig. 2d, compared to pure P123, the CABB_P123 films exhibit a notable redshift in the C–O–C stretching vibration from 1105 to 1088 cm^−1^, along with a shift in the C–H bending mode from 1251 to 1240 cm^−1^. A similar trend is observed in the CABB_PEG films (Fig. S7a), where the spectral region between 1390 and 1321 cm^−1^ shows a pronounced blue shift relative to pure PEG, indicating strong coordination between the PEG ether oxygens and Bi^3+^/Ag^+^ ions within the perovskite lattice.^31^ In contrast, no significant vibrational signals and shifts are detected in CABB_PPG (Fig. S7b), indicating no defined coordination and implying that its role is mainly steric rather than chemical. Most probably, during the washing and centrifugation steps of CABB_PPG, a substantial fraction of the hydrophobic PPG was removed, whereas the PEG in CABB_PEG tends to remain attached to the perovskite lattice due to its strong coordination with metal ions. These observations indicate that PEG segments are the principal contributors to chemical binding, highlighting their decisive role in governing polymer–metal interactions.

To further clarify which perovskite cation primarily contributes to this coordination behavior, individual precursors (CsBr, AgBr, and BiBr_3_) were mixed with P123 and analyzed. Notably, the Ag^+^-containing system induced the most intense peaks with a significant shift in the ether vibrational region (Fig. 2e), suggesting a preferential coordination of Ag^+^ with the PEG segments of P123. This indicates that in the early stage of the reaction, Ag^+^ is selectively bound to P123. Under conditions where Ag^+^ is temporarily unavailable, coordination among the polymer additive with Cs^+^, Bi^3+^, and Br^−^ preferentially forms the intermediate Cs_3_Bi_2_Br_9_ (CBB) with a layered structure of distorted [Bi_2_Br_9_]^3−^ dimers. This is demonstrated in Fig. 2f by the characteristic XRD reflections at 12.7°, 25.5°, and 27.1° observed in the respective sample collected after one minute. As shown in Fig. S8, the suspension at this stage exhibits a bright yellow color, consistent with the characteristic optical appearance of CBB. As the reaction proceeds, Ag^+^ ions are gradually released from the polymer complexes and react with Br^−^ to form [AgBr_6_]^5−^ octahedra which in their turn incorporate into the Cs_3_Bi_2_Br_9_ lattice, driving the transformation toward the final CABB phase.^32^ XRD patterns of CABB_P123 recorded after 30 and 60 minutes show progressive weakening of CBB peaks and emerging dominance of CABB reflections, confirming complete phase conversion. The final product adopts a three-dimensional double perovskite structure composed of alternating [AgBr_6_]^5−^ and [BiBr_6_]^3−^ octahedra. This process relieves lattice distortion and drives conversion to the symmetric CABB, as confirmed by the appearance of a uniform orange suspension and loss of CBB peaks^32^ in the diffractograms. This behavior aligns with the reported cation-exchange pathway from CBB to CABB, where a critical threshold of Ag^+^ incorporation is necessary to induce the full phase transition.^33,34^ Unlike pure PEG, which over-coordinates Ag^+^ and slows down the conversion resulting in the maintenance of the CBB phase as detected by XRD (Fig. S5), P123 partly contains hydrophobic PPG segments that provide steric hindrance, enabling more controlled nucleation behavior. These findings confirm that P123 acts not only as a binder but also as a spacer and crystallization modulator with chemical selectivity, bridging adjacent crystals and enhancing the mechanical cohesion of the resulting thick film.

### Device fabrication and performance

**Fig. 3:**
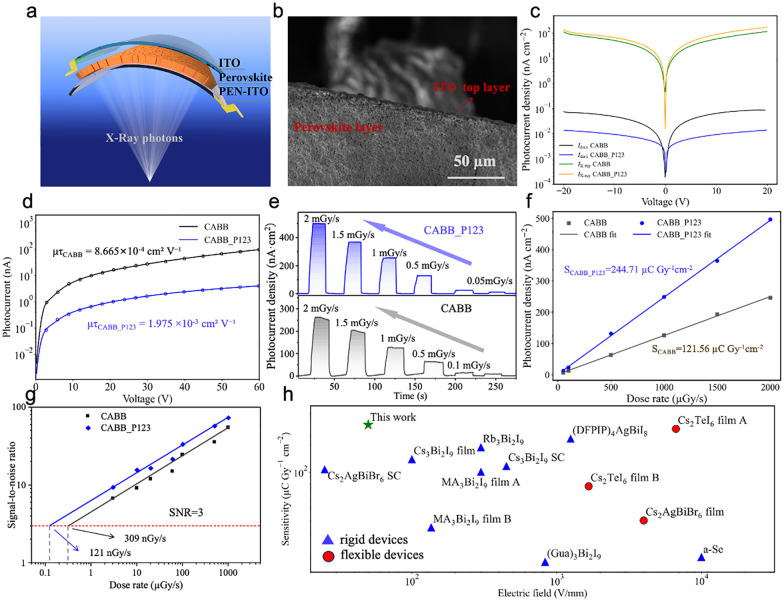
(a) Device architecture for X-ray detection measurements. (b) SEM image of the cross-section of the perovskite layer with semi-transparent ITO top electrode. (c) Dark/X-ray current density–voltage curves. (d). Carrier transport (*µτ*) fitting using the Hecht equation. (e) *I–t* response of CABB and CABB_P123 devices under X-ray irradiation with different dose rates. (f) Fitting line of photocurrent densities at different dose rates of CABB and CABB_P123 devices; error bars, correspond to the standard deviation of photocurrent density, are smaller than the symbol size. (g) Limit of detection of CABB and CABB_P123 devices. (h) Comparison of the X-ray sensitivity of rigid/flexible lead-free X-ray detectors under various electric fields.

In the low-to-medium X-ray energy range, Cs_2_AgBiBr_6_ exhibits a relatively large attenuation coefficient owing to the presence of high-*Z* elements such as Ag and Bi.^25^ Fig. S9 shows the attenuation efficiency of Cs_2_AgBiBr_6_ under 30 keV X-ray irradiation. A 100 µm-thick layer can absorb approximately 73.6% of the incident photons under 30 keV X-ray irradiation, demonstrating strong intrinsic attenuation and supporting its applicability in X-ray detectors. A film thickness of about 100 µm was selected to balance X-ray absorption and mechanical flexibility. Further increasing the film thickness led to a pronounced loss of flexibility, which can be attributed to insufficient stress relaxation in thicker layers. Based on this optimized thickness, vertical-type photodetectors were fabricated by depositing a semi-transparent ITO layer onto the film surface as the top electrode, as illustrated in Fig. 3a and b. The resulting device architecture is ITO/CABB/ITO-PEN.

Further increasing the film thickness significantly compromised the mechanical flexibility due to poor stress relaxation. Next, we fabricated vertical-type photodetectors by depositing a semi-transparent ITO on the sample surface as the top electrode, as shown in Fig. 3a and b. The resulting device architecture is ITO/CABB/ITO-PEN.

To evaluate the electrical and X-ray detection performance of the fabricated thick-film devices, current–voltage (*I*–*V*) characteristics were recorded for both CABB and CABB_P123 under dark and X-ray irradiation conditions over a bias range of −20 V to 20 V. As shown in Fig. 3c, the incorporation of Pluronic P123 into the CABB matrix effectively suppresses the dark current over the entire voltage range, while simultaneously enhancing the X-ray induced photocurrent, indicating improved detection sensitivity. To assess the intrinsic charge carrier transport, photoconductivity measurements were performed under 470 nm blue LED illumination (Fig. 3d). While X-ray based Hecht analysis has been reported for millimeter-thick single crystals, visible-light excitation is more appropriate for the present 100 µm films to ensure reliable *µτ* extraction. ^25^ The experimental data were fitted using the modified Hecht equation, a widely adopted model for evaluating carrier collection efficiency in radiation detectors:
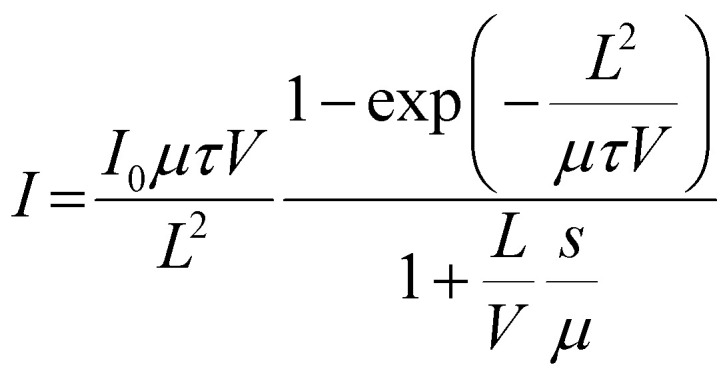
*I*(*V*) is the photocurrent at applied voltage *V*, *I*_0_ the saturated photocurrent, *L* the film thickness, *µ* the carrier mobility, *τ* the carrier lifetime, and s is the surface recombination velocity. As depicted in Fig. 3d, the CABB_P123 sample exhibits about 2.3 times higher *µτ* of 1.975 ×10^−3^ cm^2^ V^−1^, compared to 8.665 × 10^−4^ cm^2^ V^−1^ for the pristine CABB. These results indicate enhanced charge transport and reduced trap-mediated recombination due to enhanced CABB crystal quality, connectivity, and interface passivation. This was further confirmed by the X-ray detection sensitivity, which is determined using the current-density formulation:
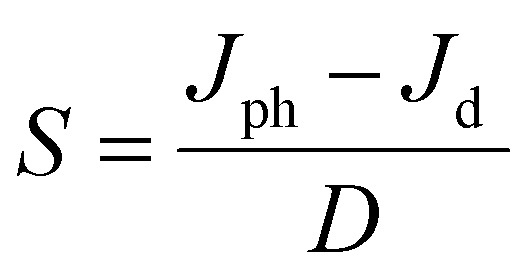
where *J*_ph_ and *J*_d_ are the photocurrent density and dark current density, respectively, and *D* is the incident X-ray dose rate.^35^ As shown in Fig. 3e, under a low applied electric field of 50V/mm, the CABB_P123 device exhibited distinct on/off switching across different dose rates and delivers a significantly enhanced X-ray sensitivity of 244.71 µC Gy^−1^ cm^−2^, more than twice that of the pristine CABB device (121.56 µC Gy^−1^ cm^−2^, Fig. 3f). The limit of detection (LoD) represents a critical performance indicator for X-ray detectors, as it directly determines the minimum radiation dose that can be safely and reliably identified during inspection. Following the IUPAC definition, the LoD corresponds to the dose rate at which the signal-to-noise ratio (SNR) is equal to 3.^36^ By performing a linear fitting, the P123-modified CABB detector exhibits a remarkably low LoD of 121 nGy s^−1^ (Fig. 3g), which is approximately three times lower than that of the pristine CABB device (309 nGy s^−1^). Both values are significantly lower than the minimum LoD required for medical diagnostic imaging (5.5 µGy s^−1^).

Fig. 3h compares the X-ray sensitivity at various electric fields of lead-free perovskite-based detectors reported in literature.^25,37–48^ Most previously reported devices require higher operating fields and exhibit lower sensitivity, while the device developed in our work simultaneously achieves mechanical flexibility and high sensitivity at a relatively low applied voltage. The temporal response is defined as the time required for the photocurrent to rise from 10% to 90% of its steady-state value, or to fall from 90% to 10% during decay.^35,49^ Owing to the enhanced carrier transport in the P123-modified films, the device exhibited a rapid switching behavior under an X-ray dose rate of 500 µ Gy s^−1^, with rise and decay times of 267 ms and 732 ms, respectively. Both were faster than those of the pristine CABB device (Fig. S10a–d). After 15 days storage, the response times of device CABB_P123 remain largely preserved (Fig. S10e–h), indicating that the incorporation of P123 effectively stabilizes charge transport pathways, in agreement with the *µτ*-product enhancement observed from Hecht-equation fitting.

**Fig. 4:**
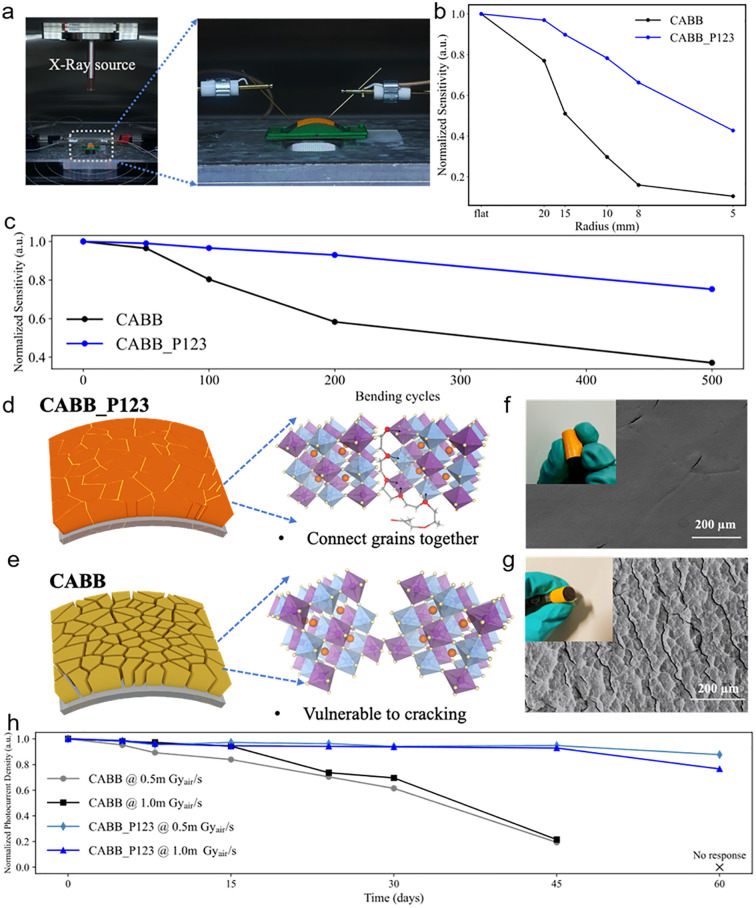
(a) Photographs of the experimental setup for *in situ* X-ray irradiation and bending tests. (b) Normalized X-ray sensitivity of sample CABB and CABB_P123 under various bending radii. (c) Normalized X-ray sensitivity of CABB and CABB_P123 devices at repeated bending cycles. (d) and (e) Schematic illustration of P123 acting as a binder to enhance grain connectivity and mechanical robustness in CABB. SEM images of the films of (f) CABB_P123 and (g) pristine CABB surface after 500 bending cycles, insets show photographs of the bent samples. (h) Normalized photocurrent density measured under 0.5 and 1.0 mGy s^−1^ over 60 days evaluating the long-term stability of CABB and CABB_P123 devices.

Beyond detection performance, the mechanical flexibility of the devices was systematically evaluated to assess their robustness under mechanical deformation. The X-ray response of both pristine CABB and CABB_P123 films on flexible ITO/PET substrates was measured under various bending radii. As illustrated in Fig. 4a, the devices were placed on a bending stage with micromanipulator probes in contact, allowing *in situ* electrical measurements under defined mechanical deformation. X-ray irradiation was applied vertically during bending, while current density was continuously recorded to assess device response. Sensitivity variation was evaluated as a function of dose rate at bending radii ranging from 20 mm to 5 mm. As shown in Fig. 4b, both devices exhibited a gradual decrease in sensitivity with decreasing bending radius, indicating strain-induced degradation. However, the CABB_P123 device consistently delivered higher current densities across all dose rates and bending conditions (Fig. 11a), maintaining over 70% of its initial sensitivity at a bending radius of 8 mm. This improved performance is attributed to the incorporation of P123, which effectively relieves residual stress through crosslinking at grain boundaries, thereby enhancing mechanical flexibility. In contrast, the pristine CABB device showed a more pronounced decline (Fig. S11b), confirming the enhanced mechanical resilience by the polymer-assisted crystallization strategy. In addition, the dark current density of both devices was also evaluated under different bending radii, as shown in Fig. S12. The CABB_P123 device exhibits highly reproducible dark-current curves across all bending conditions, with only minor variations at a bending radius of 5 mm. In contrast, the pristine CABB device displays a pronounced increase in dark current and noticeable fluctuations when subjected to reduced bending radii. This comparison further confirms that the incorporation of P123 stabilizes grain connectivity and effectively suppresses strain-induced leakage pathways, thereby allowing the thick CABB films to retain low-dark-current operation under mechanical deformation.

As shown in Fig. S13a and b, both CABB and CABB_P123 devices were subjected to cyclic bending, and the normalized X-ray sensitivity was recorded as shown in Fig. 4c. The CABB_P123 device retained over 95% of its initial sensitivity after 100 cycles and approximately 80% after 500 cycles, demonstrating excellent fatigue resistance. In contrast, the pristine CABB device showed a rapid degradation, with sensitivity dropping below 60% after 200 cycles and falling below 40% after 500 cycles (Fig. 4c). As schematically illustrated in Fig. 4d and e, the incorporation of P123 enhances mechanical integrity by bridging adjacent grains and relieving grain boundary stress. In contrast, the pristine CABB film, due to the intrinsic brittleness of the all-inorganic perovskite structure, lacks such reinforcement and is prone to crack formation under repeated bending. The mechanical behavior of CABB and CABB_P123 films under bending was further evaluated through device photographs and SEM images (Fig. 4f and g). After 500 bending cycles, the pristine CABB film exhibited obvious surface damage, whereas the P123-modified film maintained a more continuous and compact morphology, confirming its superior mechanical robustness.

The long-term storage stability of the devices was assessed by monitoring their photocurrent response under ambient air, dark, and room-temperature conditions over a 60-day period (Fig. S14). Fig. 4h presents the time-resolved photocurrent responses of pristine CABB and P123-modified devices under X-ray irradiation at 500 µGy s^−1^ and 1000 µGy s^−1^. The CABB_P123 device exhibited highly stable operation during the first 30 days, it preserved over 90% of its initial photocurrent amplitude. In contrast, the pristine CABB device had already lost more than 60% of its initial response, accompanied by increasing dark current and reduced pulse amplitude over time. After 60 days, the CABB_P123 device still maintained consistent photocurrent output under both irradiations. In comparison, the pristine CABB device degraded severely, maintaining <20% of its original response and showing markedly increased dark current and reduced pulse definition. In addition, we also performed the operational stability under continuous X-ray exposure with a dose rate of for approximately 5000 s. As shown in Fig. S15, throughout the entire process, the device of CABB_P123 maintains an ultra-stable current, indicating a stable charge generation and collection process under sustained X-ray excitation. These results further confirm the excellent operational robustness and long-term stability of the CABB_P123 device under practical working conditions.

## Conclusions

In summary, we applied an amphiphilic block copolymer P123 to enhance crystallization, crystal quality, and long-term mechanical and environmental stability of microcrystalline CABB double perovskite. The PEG segments coordinate with free Ag^+^ cations and regulate the crystallization from layered CBB into the stable CABB double perovskite structure, mediated by the hydrophobic PPG segments that in their turn suppress excessive PEG-Ag^+^ coordination. Additionally, P123 serves as a soft binder smoothly connecting adjacent microcrystals and contributing to the overall stability and performance of the corresponding thick film devices. Devices based on CABB_P123 delivered a significantly enhanced X-ray sensitivity of 244.71 µC Gy^−1^ cm^−2^ and low LoD of 121 nGy s^−1^ under low applied voltage field, significantly surpassing the pristine CABB device due to enhanced charge carrier transport across the crystallite interfaces. Moreover, the CABB_P123 device exhibited outstanding mechanical durability and ambient stability, retaining 80% sensitivity after 500 bending cycles and maintaining stable X-ray response over 60 days while pristine devices rapidly degraded. This approach provides a scalable route toward flexible, high-performance radiation detectors based on lead-free, all-inorganic thick-film perovskites and opens new directions for multifunctional polymer design in next-generation perovskite optoelectronics.

## Experimental

### Materials

Cesium bromide (CsBr 99.999%, Sigma Aldrich), bismuth bromide (BiBr3, >98%, Sigma Aldrich), silver bromide (AgBr, 99%, Sigma Aldrich), poly(ethylene glycol)-*block*-poly(propylene glycol)-*block*-poly(ethylene glycol) (P123) (Sigma Aldrich), poly(ethylene glycol) (Sigma Aldrich), poly(propylene glycol) (Sigma Aldrich), were used as precursor materials. Dimethyl sulfoxide (DMSO, ≥99.5% Sigma Aldrich) and 2-propanol (IPA, 99.99%, Sigma Aldrich) were applied as the solution and anti-solvent. All the chemicals were used as received without any further purification.

### Synthesis methods

The Cs_2_AgBiBr_6_ (CABB) precursor solution was prepared by dissolving 0.426 g (2 mmol) of CsBr, 0.449 g (1 mmol) of BiBr_3_, and 0.188 g (1 mmol) of AgBr in 10 ml of dimethyl sulfoxide (DMSO) under continuous stirring at 60 °C for 2 hours. P123 was first dissolved in DMSO and subsequently introduced into the precursor solution. To investigate the effect of polymer concentration, precursor solutions containing varying amounts of P123 (1, 5, 10, 20, and 30 wt%) were prepared and stirred at 60 °C for 2 hours. P123 at a mass concentration of 10 wt% was selected to conduct further experiments.

Subsequently, 5 ml of each precursor solution (with and without P123) was added dropwise into 35 ml of isopropanol under vigorous stirring at 60 °C for 2 hours. This anti-solvent process induced the precipitation of CABB and CABB_P123 microcrystals, and the slurry precipitates were collected *via* centrifugation at 4000 rpm for 3 minutes. Subsequently, the CABB or CABB_P123 slurries were deposited onto PEN/ITO substrates maintained at 40 °C by drop casting in a template with an area of 1.5 × 1.5 cm^2^ and controlled to 100 µm by controlling the drop amount, followed by soft pressing with 0.1 MPa pressure applied to the perovskite film to ensure film uniformity and adhesion.

### Device fabrication and mechanical flexibility test

After achieving the desired thick films, a uniform top electrode was deposited by spin-coating an ITO nanoparticle suspension onto the film surface. The final device was dried under ambient conditions. The active area of each device was defined as 1.5 × 1.5 cm^2^, determined by the overlap between the top and bottom ITO electrodes. The LoD was derived from the fitting line with an SNR of 3. Fixed-radius bending tests were conducted by fixing the devices onto a custom-designed arc-shaped mold with bending radii of 5, 8, 10, 15, and 20 mm. The X-ray sensitivity of each device was measured under the corresponding bending condition and normalized to its flat-state value to assess the sensitivity retention. In addition, mechanical durability was evaluated by cyclic bending tests, in which the devices were subjected to repeated bending cycles under a constant bending radius of 10 mm. The normalized sensitivity was recorded at different cycle numbers to determine the long-term mechanical stability of the devices.

## Conflicts of interest

All authors declared no financial/commercial conflicts of interest.

## Supplementary Material

TC-014-D6TC00075D-s001

## Data Availability

The data supporting this article have been included as part of the supplementary information (SI). Supplementary information is available. See DOI: https://doi.org/10.1039/d6tc00075d.
