# Supplementary material for: Bifunctional polymer assisted growth of crack-free thick perovskite films for flexible X-ray detection
Source: J Mater Chem C Mater. 2026 Mar 20;14(19):8077–84. doi: 10.1039/d6tc00075d (PMC13016186; doi:10.1039/d6tc00075d)
Supplement: TC-014-D6TC00075D-s001 [file TC-014-D6TC00075D-s001.pdf]

Supplementary Information (SI) for Journal of Materials Chemistry C.
This journal is © The Royal Society of Chemistry 2026

Supporting Information

Bifunctional Polymer Assisted Growth of Crack-free Thick Perovskite Films for Flexible X-ray Detection

Qianrui Li[a], Donato Valli [a,c], Roel Vanden Brande[a], Giorgia Rizzi[a], Johan Hofkens[a,d], Wei Qu[a,e], and Elke Debroye[a,b]**

[a] Department of Chemistry, KU Leuven, Celestijnenlaan 200F, 3001, Heverlee, Belgium

[b] State Key Laboratory of Advanced Fiber Materials, Donghua University, Shanghai 201620, People's Republic of China

[c] Institute of Supramolecular Science and Engineering (ISIS), Strasbourg, Grand Est, France

[d] Max Planck Institute for Polymer Research, 55128 Mainz, Germany

[e] School of Optical and Electronic Information, Suzhou City University & Suzhou Key Laboratory of Biophotonics, Suzhou, 215104, China

E-mail: elke.debroye@kuleuven.be

Fig. S1 a) XRD patterns of CABB films with varying P123 concentrations. Diffraction peaks of b) [200] plane and c) [400] plane of CABB with varying P123 concentrations. d) Full width at half maximum (FWHM) of the [400] diffraction peak. 5

Fig. S2 SEM images of powder CABB reacted with a)1%, b) 5%, c) 20%, d) 30% P123 6

Fig. S3 a) UV-Vis spectra and b) corresponding Tauc plots of CABB with varying P123 concentrations. 7

Fig. S4 SEM images of a) CABB_PEG and b) CABB_PPG powder. 8

Fig. S5 XRD patterns of CABB, CABB_P123, CABB_PEG and CABB_PPG. 9

Fig. S6 SEM images of film CABB_PPG with cracks 10

Fig. S8 Visual evolution of CABB suspensions reacted with P123, PEG and PPG. 12

Fig. S9 X-Ray attenuation efficiency 13

Fig.S10 Temporal response 14

Fig. S11 Photocurrent density of a) CABB_P123 and b) CABB devices under various bending radii. 15

Fig. S12 Dark current density of CABB_P123 and CABB devices at different bending radius. .16

Fig. S13 Photocurrent density of a) CABB_P123 and b) CABB devices over repeated bending cycles. 17

Fig. S14 The X-ray photocurrent density of CABB_P123 and CABB devices irradiated by 0.5 and 1.0 m Gy_{air}/s over 60 days under a bias of 50 V mm^{-1}. 18

Fig. S15 Operational stability of CABB_P123 and CABB devices under continuous irradiation. 19

Characterization Techniques

Powder X-ray Diffraction (XRD)

XRD patterns were recorded with a Malvern Panalytical Empyrean diffractometer in transmission mode by using a pixcel3d solid-state detector with a Cu anode. The diffractogram was recorded in the range of 2θ between 5 and 45°.

Scanning electron microscopy (SEM)

SEM was used to obtain images of the crystals. They were recorded on a FEI-Q FEG250 instrument.

UV-Vis Diffuse Reflectance Spectroscopy

Diffuse-reflectance spectroscopy data were recorded on a Lambda 950 UV–vis spectrophotometer (PerkinElmer) using $BaSO_4$ powder as a white reference. The resulting diffused reflectance (R) was converted to $F(R) = (1 - R)^2/2R$ by using the Kubelka–Munk function. The indirect bandgap values were extracted by means of Tauc plot analysis.

Fourier-Transform Infrared Spectroscopy

Attenuated total reflection–FTIR (ATR–FTIR) spectra were recorded on a Varian 670 FTIR spectrometer equipped with a VeeMAXTM III accessory (PikeTech)

X-ray Irradiation Setup

The X-ray related measurements were performed using an Xstrahl Life Sciences cabinet irradiator equipped with a tungsten anode X-ray tube. The tube voltage was set to 30 keV to generate X-ray photons, and the sample was positioned at a fixed distance of 0.3 m from the source. All X-ray measurements were conducted in air and at room temperature. The X-ray dose rate was precisely controlled by adjusting the tube current while keeping the voltage constant. Real-time dose measurements were acquired using a PTW UNIDOS electrometer (PTW-Freiburg, Germany) connected to a calibrated ionization chamber. During the experiments, the dose rate typically ranged from 5000 $\mu Gy\ s^{-1}$ to 3 $\mu Gy\ s^{-1}$, depending on the irradiation conditions. For each dose-rate value, the measurements were repeated three times ($n = 3$), and the average photocurrent density was used for plotting. The corresponding error bars, calculated as the standard deviation

of these repeated measurements, are significantly smaller than the symbol size and therefore not visible in the main plot.

Electrical measurements were carried out using a Keithley 2400 source meter, which provided a stable bias voltage across the sample and simultaneously recorded the photocurrent. All measurements were conducted in the dark to eliminate ambient light interference and to highlight the intrinsic X-ray detection capability of the perovskite-based devices.

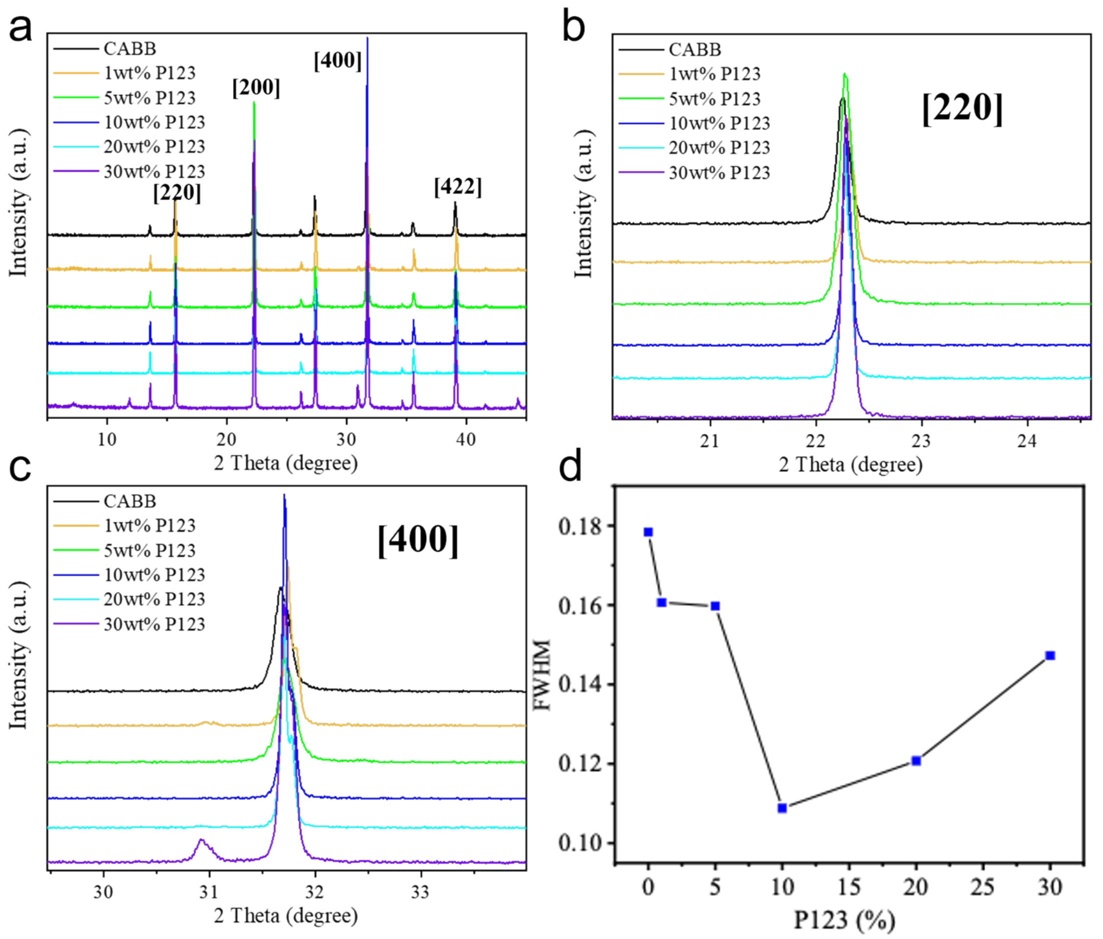

Fig. S1 a) XRD patterns of CABB films with varying P123 concentrations. Diffraction peaks of b) [200] plane and c) [400] plane of CABB with varying P123 concentrations. d) Full width at half maximum (FWHM) of the [400] diffraction peak.

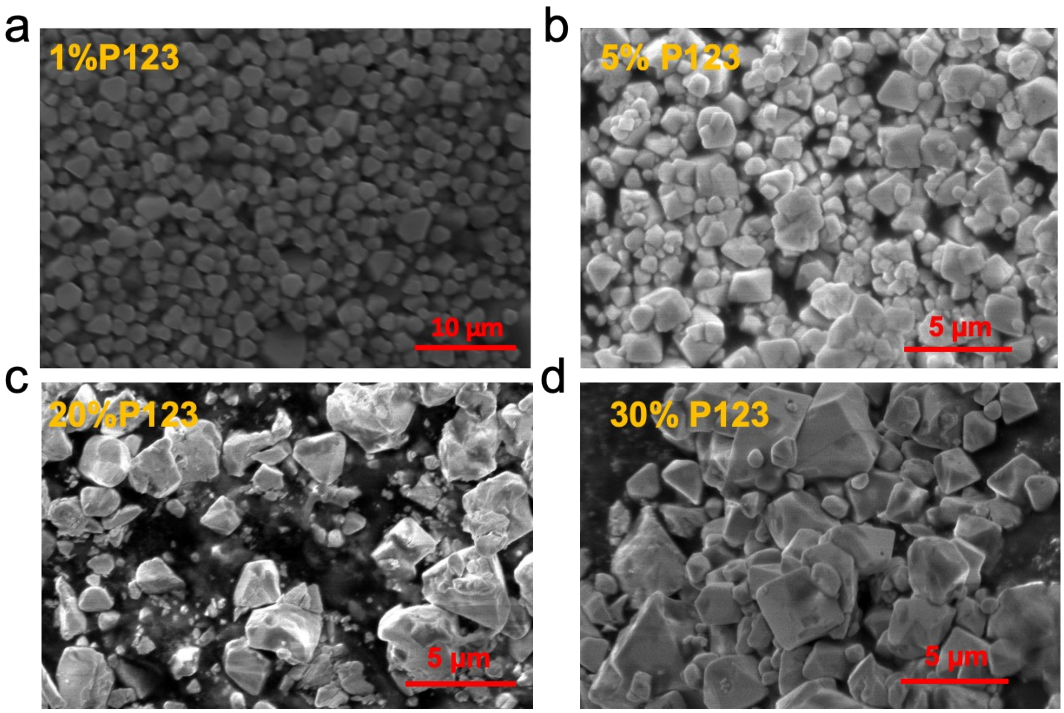

Fig. S2 SEM images of powder CABB reacted with a)1%, b) 5%, c) 20%, d) 30% P123

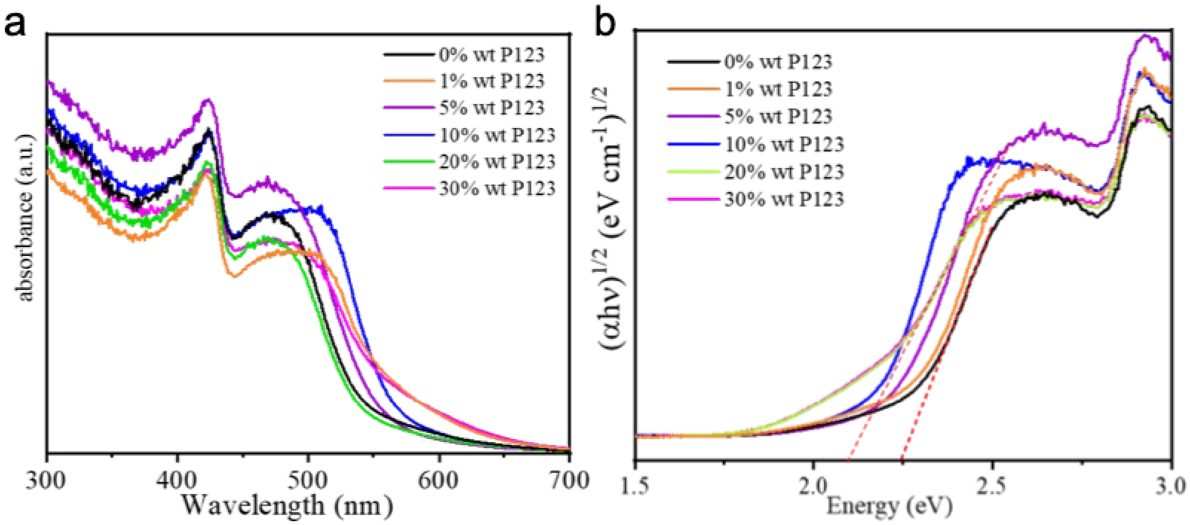

Fig. S3 a) UV-Vis spectra and b) corresponding Tauc plots of CABB with varying P123 concentrations.

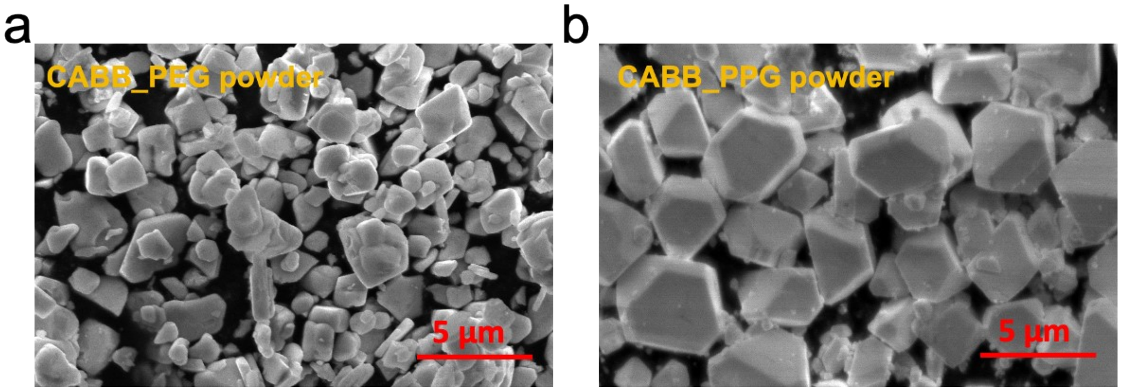

Fig. S4 SEM images of a) CABB_PEG and b) CABB_PPG powder.

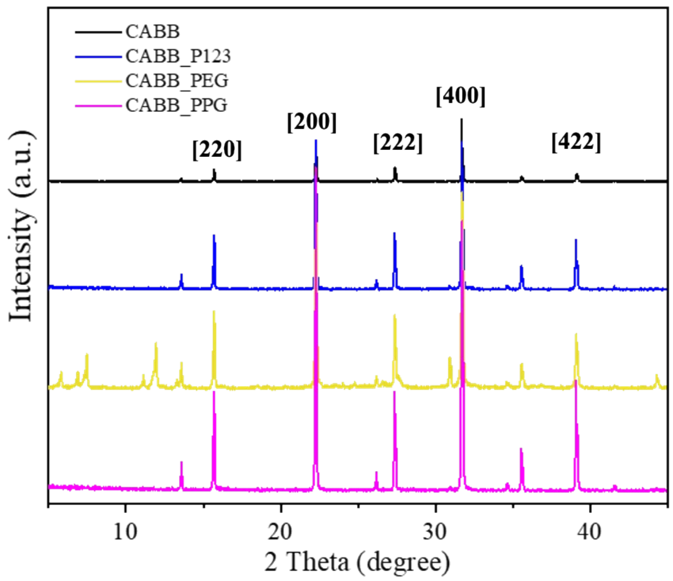

Fig. S5 XRD patterns of CABB, CABB_P123, CABB_PEG and CABB_PPG.

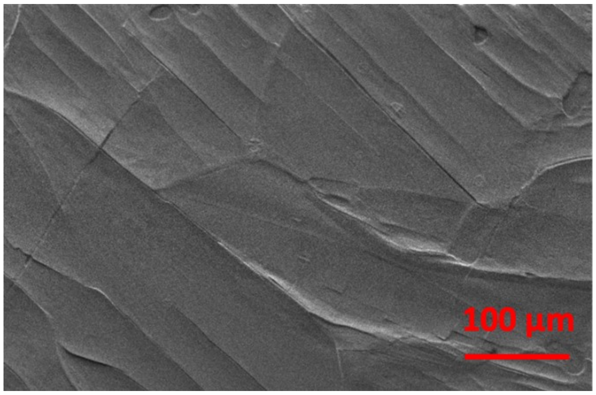

Fig. S6 SEM image of film CABB_PPG showing cracks after 100 cycles of bending over a bending radius of 10mm.

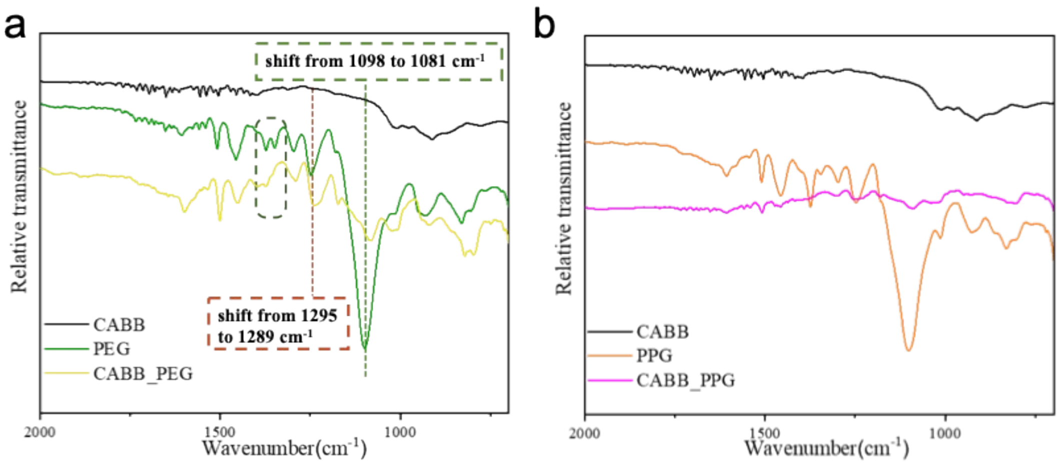

Fig. S7 FT-IR spectra of CABB_PEG and CABB_PPG.

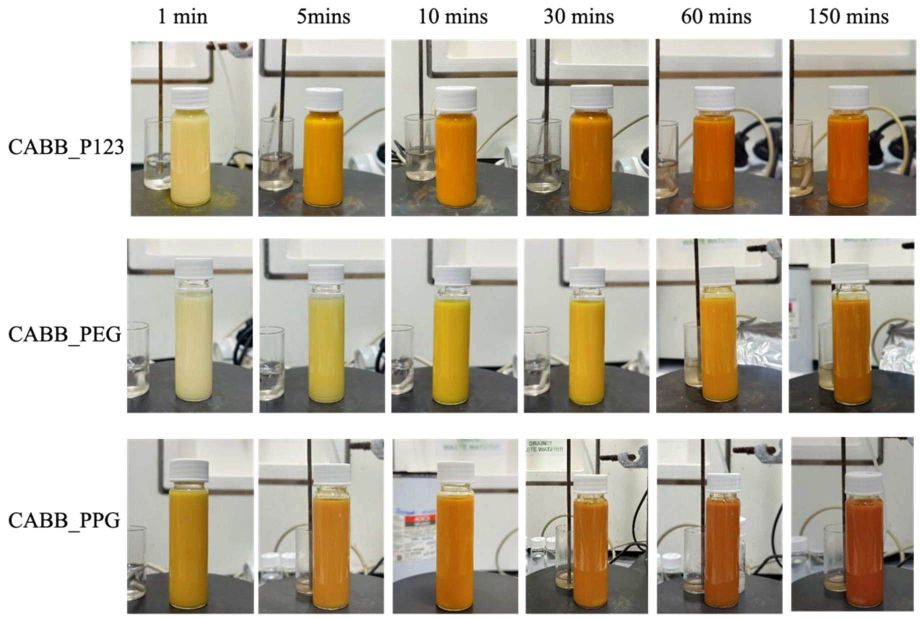

Fig. S8 Visual evolution of CABB suspensions reacted with P123, PEG and PPG.

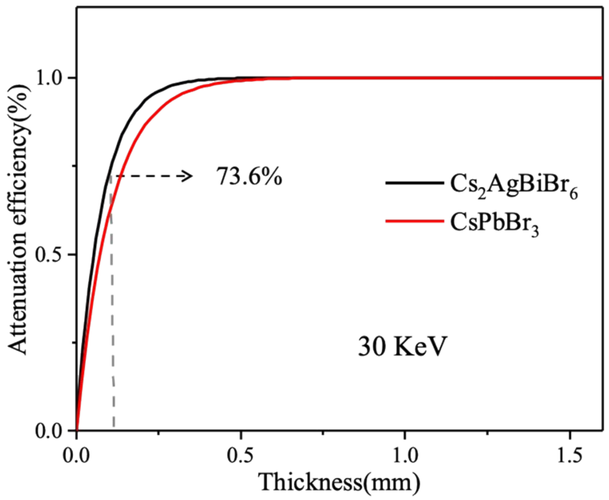

Fig. S9 a) X-ray attenuation efficiency of $Cs_2AgBiBr_6$ and $CsPbBr_3$ to 30 KeV X-ray photons versus layer thickness.

The curve was obtained by calculating the X-ray attenuation coefficients under X-ray photon energy of 30 keV and fitting the exponential attenuation law, which matches our experimental conditions.

$$I_{(x)}=I_0\ e^{-\mu x}$$

Where I_0 is incident intensity, $I_{(x)}$ transmitted intensity, μ is linear attenuation coefficient, x is material thickness. For comparison, we also included the calculated attenuation behavior of a standard perovskite ($CsPbBr_3$) under the same conditions.

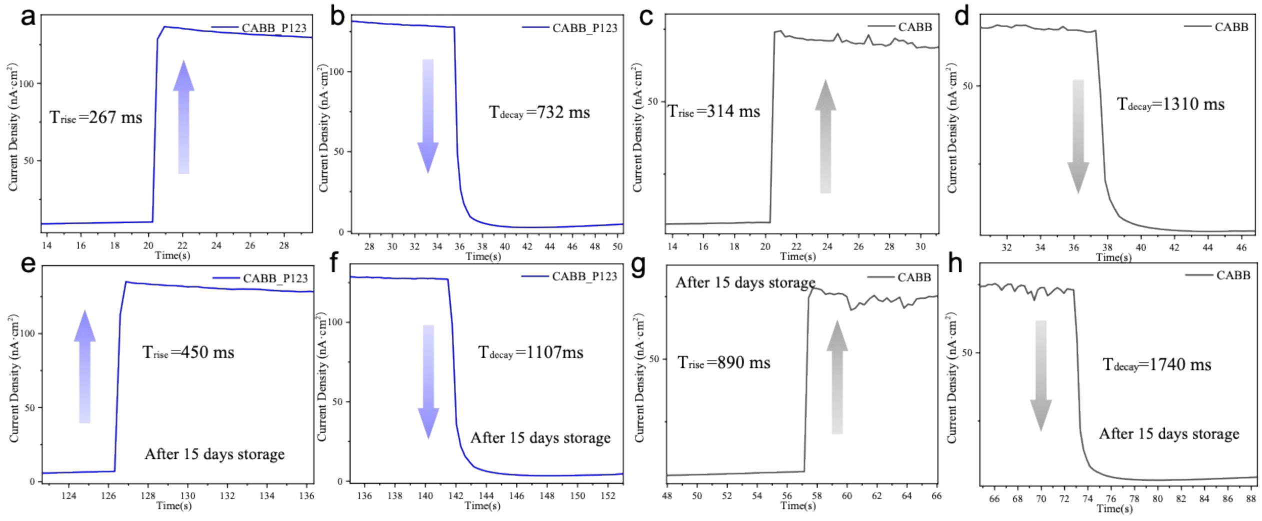

Fig.S10 Temporal response of a-b) CABB_P123 and c-d) CABB devices, and e-f) CABB_P123 after 15 days storage and g-h) CABB devices after 15 days storage under an X-ray dose rate of 500 μGy s^{-1}.

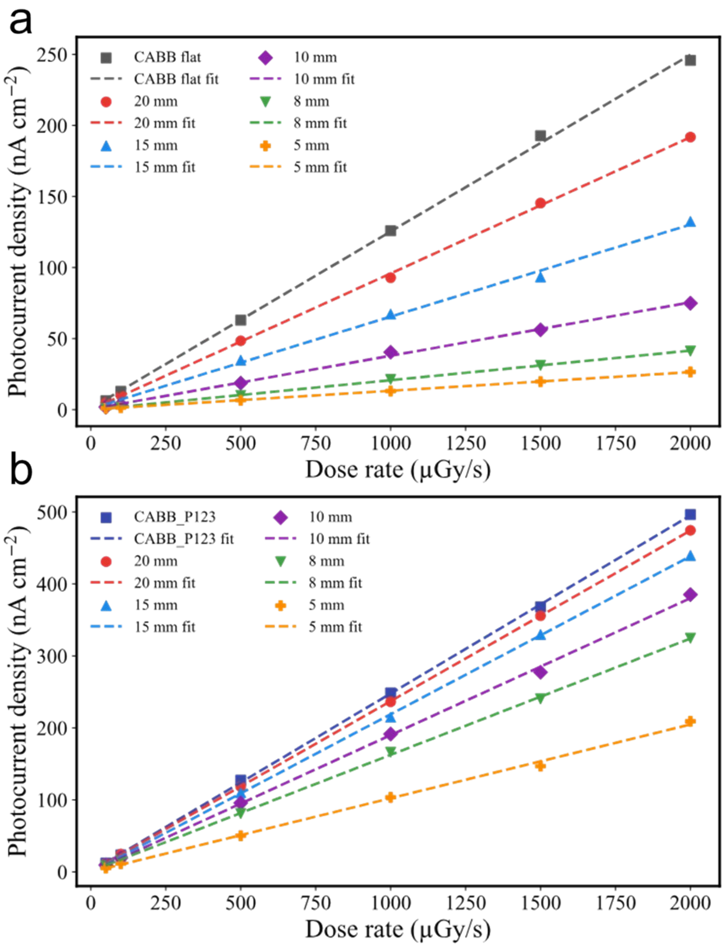

Fig. S11 Photocurrent density of a) CABB_P123 and b) CABB devices under various bending radii.

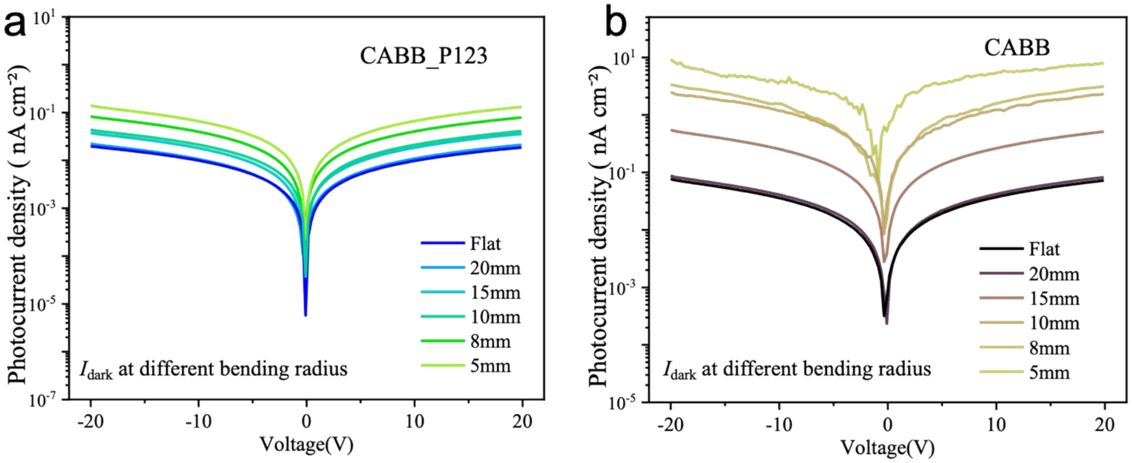

Fig. S12 Dark current density of CABB_P123 and CABB devices at different bending radii and when restored to the flat condition after the bending tests.

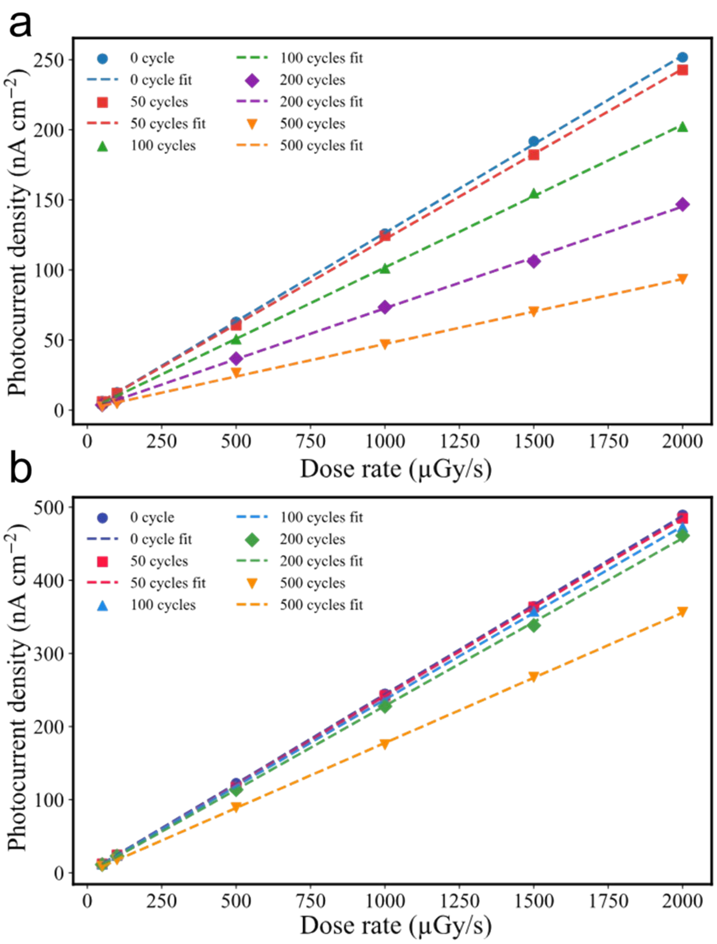

Fig. S13 Photocurrent density of a) CABB_P123 and b) CABB devices over repeated bending cycles.

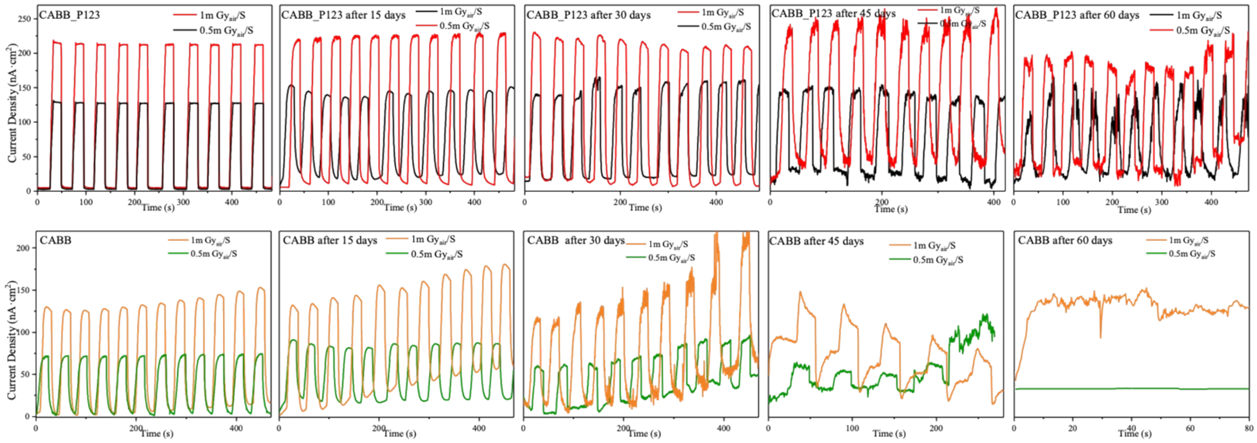

Fig. S14 The X-ray photocurrent density of CABB_P123 and CABB devices irradiated by 0.5 and 1.0 m Gy$_{air}$/s over 60 days under a bias of 50 V mm^{-1}.

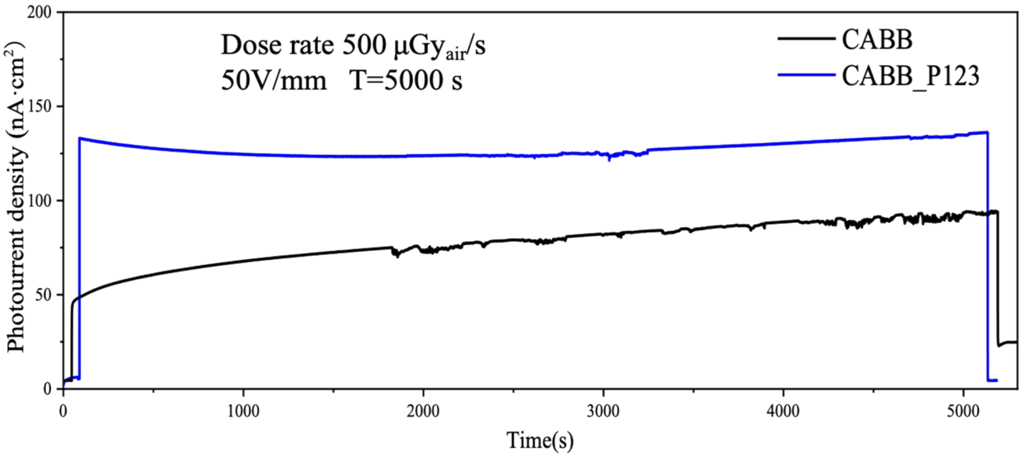

Fig. S15 Operational stability of CABB_P123 and CABB devices under continuous irradiation.
